# P-571. Exploring the Dengue Vaccination Intention among Adults from Regions with Medium and High Incidence of Dengue in Peru

**DOI:** 10.1093/ofid/ofaf695.786

**Published:** 2026-01-11

**Authors:** Jose A Gonzales-Zamora, Julieta M Araoz-Salinas, Carlos Quispe-Vicuña, Anderson Soriano-Moreno, Brando Ortiz-Saavedra, Martin E Reategui-Garcia, Gabriel A Aquino Sandoval, Wagner Rios-Garcia, Ruth Ramon-Tapia, Allison N Ortiz-Pardo, Noelia Morocho-Alburqueque, Silvia Tipe-Gonzales, Jose M Rios-Osorio, Jorge Alave

**Affiliations:** Peruvian American Medical Society, Albuquerque, NewMexico; Peruvian American Medical Society, Albuquerque, NewMexico; Red de Eficacia Clínica y Sanitaria (REDECS), Lima, Perú, Lima, Lima, Peru; Unidad de Investigación Clínica y Epidemiológica, Escuela de Medicina, Universidad Peruana Unión, Lima, Lima, Peru; Universidad Nacional de San Agustín de Arequipa, Arequipa, Arequipa, Peru; Universidad Nacional de la Amazonia Peruana, Iquitos, Loreto, Peru; Universidad Nacional de la Amazonía Peruana, Iquitos, Loreto, Perú, Iquitos, Loreto, Peru; Universidad Nacional San Luis Gonzaga, Ica, Perú., Pisco, Ica, Peru; University of Miami/Jackson Memorial Hospital - - Miami, FL, GLENDALE, New York; PERUVIAN AMERICAN MEDICAL SOCIETY, Lima, Lima, Peru; Universidad Nacional de Piura, Piura, Piura, Peru; Universidad peruana Cayetano Heredia, lima, Lima, Peru; Universidad Nacional Federico Villarreal, Lima, Lima, Peru; Universidad Peruana Union, Lurigancho Chosica, Lima, Peru

## Abstract

**Background:**

Among the several interventions to prevent dengue, vaccination has emerged as one of the most promising tools. The TAK-003 vaccine was introduced in Peru in November 2024, targeting children from 10 to 14 years. However, it is expected that this vaccine will be offered to adults soon; for this reason, it is important to evaluate the perceptions and intention to get vaccinated in this population.

**Figure 1:**
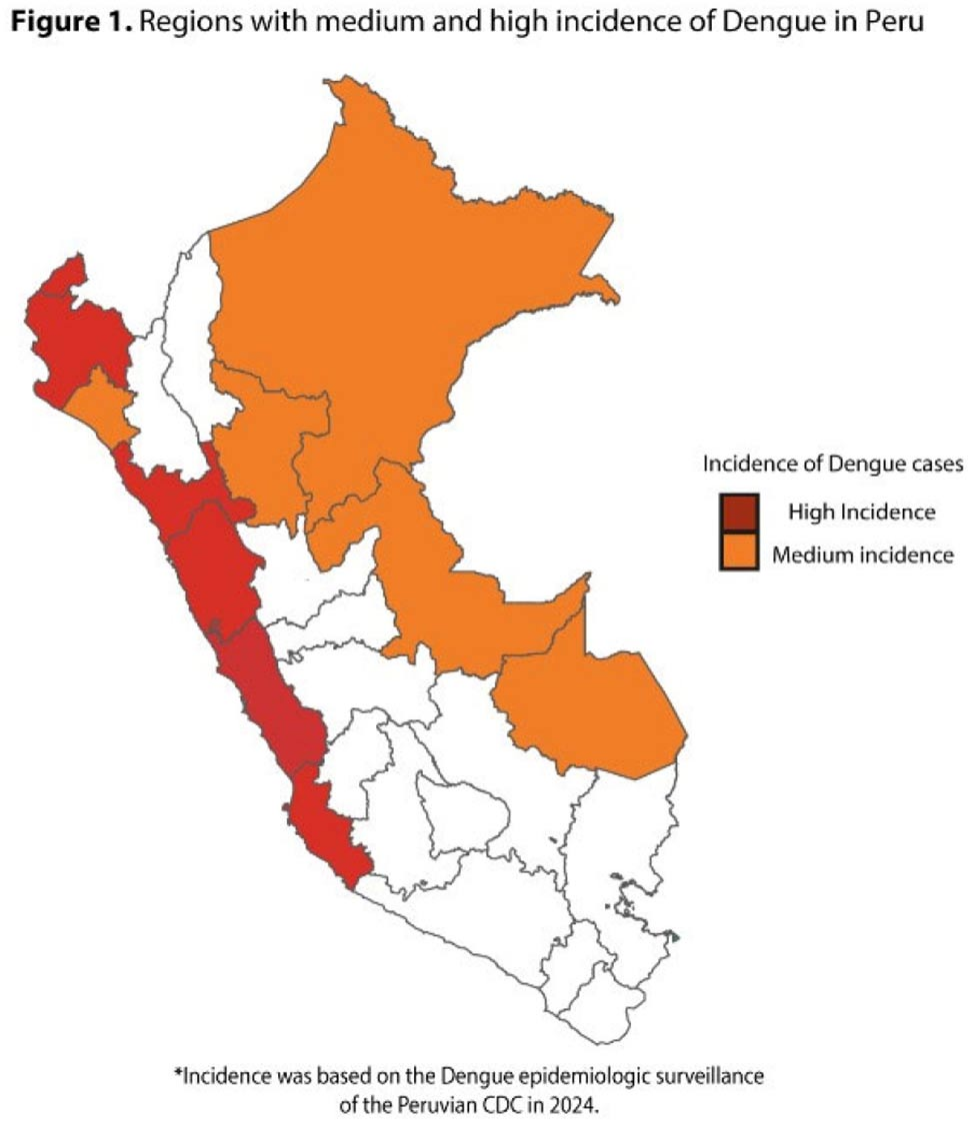
Regions with Medium and High Incidence of Dengue Fever in Peru

**Figure 2:**
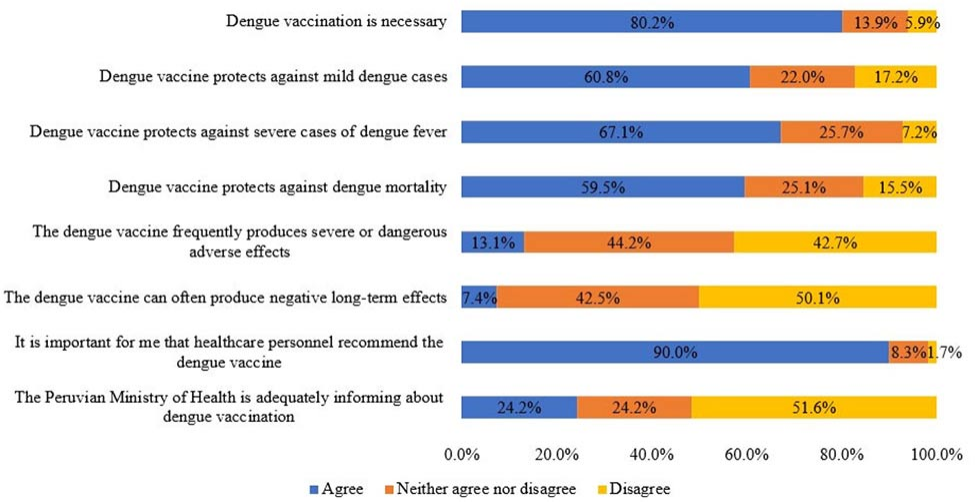
Perceptions regarding Dengue Vaccine among Adults

**Methods:**

We conducted an analytical cross-sectional study based on an online survey administered from March 22 to April 13, 2025. We evaluated the perceptions and vaccination intent among Peruvian adults from medium and high dengue incidence regions (Figure 1). To determine the factors associated with the intention to get vaccinated, we calculated crude (cPR) and adjusted (aPR) prevalence ratios with 95% confidence intervals (95% CI) using a Poisson regression model with robust variance.

**Table 1:**
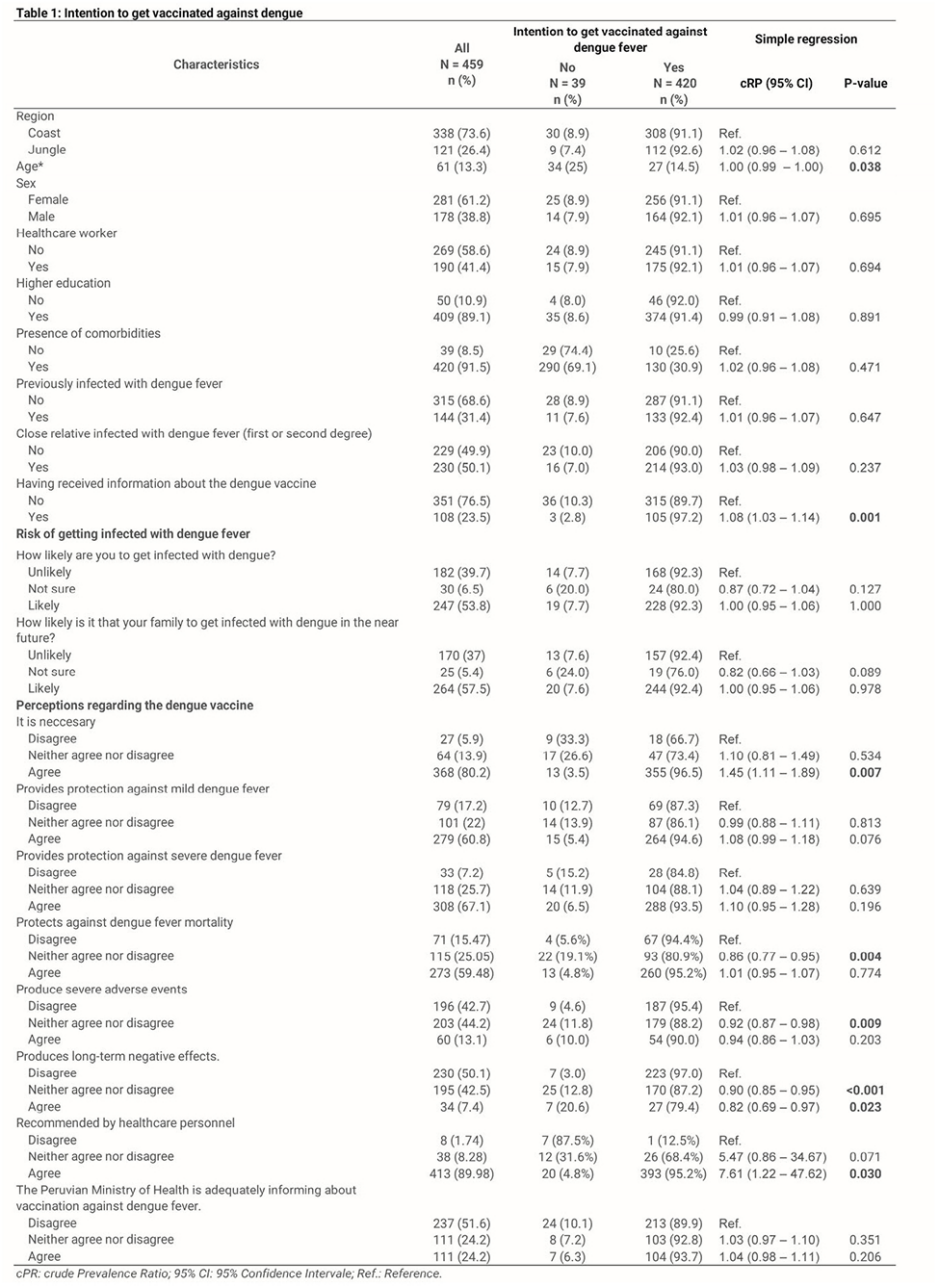
Comparison of characteristics between adults with intention to get vaccinated and those with no intention.

**Results:**

We included 459 participants (median age, 28 years; 61.2% female), 73.6% living in the Coast of Peru and 26.3% from the Jungle. Most participants (91.5%) intended to get vaccinated against dengue. Regarding perceptions, 80.2% believed the dengue vaccine was necessary, and 67.1% thought it prevented severe cases. Approximately 60% of respondents believed that the vaccine was protective against mild dengue and mortality, and only 13.1% believed it caused severe adverse events. Additionally, only 24.2% felt the Peruvian Ministry of Health was adequately informed about the vaccine (Figure 2). In the bivariate analysis, the intention to get vaccinated was significantly higher among younger participants (27 vs 34 years, p=0.018), in those who had received information about the dengue vaccine (p=0.001), in respondents who perceived the vaccine as necessary (p=0.007), and those who believed it was important that a healthcare professional recommended it (p=0.03) (Table 1).

**Conclusion:**

Most adults intend to get vaccinated against dengue in Peru. Overall, there was a positive attitude towards the vaccine. Our findings revealed that access to information significantly influenced the vaccination intention; for this reason, we believe that educational campaigns should be prioritized to enhance vaccine acceptance.

**Disclosures:**

All Authors: No reported disclosures

